# Particular etiology in a case of peripheral tetraparesis

**DOI:** 10.1002/ccr3.2157

**Published:** 2019-04-16

**Authors:** Gabriela S. Gheorghe, Andreea S. Hodorogea, Andrei C. D. Gheorghe, Ondin I. R. Zaharia, Ovidiu Neacsu, Ioan Tiberiu Nanea, Ana Ciobanu

**Affiliations:** ^1^ University of Medicine and Pharmacy Carol Davila Bucharest Romania

**Keywords:** beta thalassemia minor, hydroxybilane synthetase, nonprofessional lead poisoning, peripheral tetraparesis, porphyria

## Abstract

Lead intoxication is a rare but potentially fatal disease without appropriate intervention. The diagnosis is often difficult because of various organs involvement. We report the case of nonprofessional lead intoxication manifested by tetraparesis, severe anemia, and hemolysis in a patient having also unknown beta thalassemia minor.

## 1 INTRODUCTION

Nonprofessional lead intoxication in adults is rare; nevertheless, it may represent a fatal condition if unrecognized. Clinical and laboratory findings may be ambiguous, suggesting diseases like porphyries, hemolytic anemia, or a variety of digestive and neurological disorders. Our case illustrates the difficulties of diagnosis of lead intoxication in a patient presenting with rapid progressive tetraparesis associated with apparently unrelated clinical symptoms and nonspecific hematological tests.

## 2 CASE REPORT

We present the case of a 45‐year‐old male patient, a 14 pack‐years smoker, without significant medical history, a former worker in a coalmine, retired for 5 years. He was admitted in a hospital emergency department after 5 days of incoercible vomiting, epigastric pain, lumbar pain irradiated toward the base of the thorax, followed by muscular pain and gradual decrease in strength in upper and lower limbs.

Physical exam showed skin pallor and no organomegaly, no cutaneous bleeding, cardiovascular, or respiratory pathological changes. The neurological exam established the diagnosis of peripheral proximal tetraparesis, predominantly in the upper limbs with preserved sensitivity. These findings demanded urgent running of biological tests.

Blood tests showed severe microcytic, hypochromic anemia, increased serum iron level, serum ferritin level, and transferrin saturation coefficient and normal corrected reticulocytes count. The white blood cell and thrombocytes counts were normal (Table 1).

**Table 1:** Initial blood tests

Test	Values	Reference intervals
Red blood cells (×10^6^/μL)	2.72	3.8‐6.5
Hemoglobin (g/dL)	5.52	11.50‐17.00
Hematocrit (%)	18.4	37‐54
Mean corpuscular volume (fL)	67.8	80‐100
Mean corpuscular hemoglobin (pg)	20.3	27‐32
Mean corpuscular hemoglobin concentration (g/dL)	30.0	32‐36
Reticulocytes (%)	13.08	0‐2.5
Corrected reticulocytes Count (%)	2.29	0‐2.5
Absolute reticulocytes count (×10^6^/μL)	0.35	0‐1
Leukocytes (×10^3^/μL)	9.57	4‐10
Neutrophils (×10^3^/μL)	5.28	2‐7.5
Lymphocytes (×10^3^/μL)	3.55	1‐4
Monocytes (×10^3^/μL)	0.54	0.2‐1
Eosinophils (×10^3^/μL)	1.19	0‐0.05
Basophils (×10^3^/μL)	0.09	0‐0.2
Neutrophils (%)	55.2	45‐65
Lymphocytes (%)	37.1	25‐32
Monocytes (%)	5.62	4‐8
Eosinophils (%)	1.19	0‐5
Basophils (%)	0.91	0‐1
Thrombocytes (×10^3^/μL)	273	150‐400
Serum iron levels (μg/dL)	199.7	60‐170
Ferritin (ng/mL)	883.73	21.81‐274.66
Transferrin (mg/dL)	189	174‐364
Transferrin saturation (%)	57	15‐45
Haptoglobin (g/L)	1.13	0.3‐2

The peripheral blood smear showed anisocytosis, hypochromic red blood cells, red blood cells with basophilic granules, droplet red blood cells, and rare ovalocytes. Direct and indirect Coombs tests were negative. The bone marrow aspirate showed 46% sideroblasts, 42% ringed sideroblasts, 2+/3+ macrophages, suggesting the diagnostic of erythroid hyperplasia and sideroblastic anemia.

The remainder of the blood chemistry tests that were carried out initially showed hyperbilirubinemia with increased indirect bilirubin, inflammatory syndrome, hepatocytolysis, hepatic cholestasis, and normal renal function (Table 2).

**Table 2:** Initial blood chemistry

Test	Values	Reference intervals
Erythrocytes sedimentation rate (mm/1h)	50	1‐10 (men)
Total bilirubin (mg/dL)	2.3	0.2‐1.2
Indirect bilirubin (mg/dL)	1.47	0‐0.5
Direct bilirubin (mg/dL)	0.83	0‐0.5
Gamma‐glutamyl transpeptidase (U/L)	150	12‐64
Alkaline phosphatase (U/L)	102	40‐150
Aspartate aminotransferase (U/L)	66	5‐34
Alanine aminotransferase (U/L)	117	5‐55
Glycemia (mg/dL)	93	70‐99
Creatine kinase (U/L)	27	30‐200
Creatine kinase MB (U/L)	21	0‐24
Lactate dehydrogenase (U/L)	184	125‐220
Creatinine (mg/dL)	1.02	0.72‐1.25
Cholesterol (mg/dL)	158	<200
Serum K^+^ (mmol/L)	4.3	3.5‐5.1
Serum Na^+^ (mmol/L)	135	136‐145
Thyroid stimulating hormone (µU/mL)	1.21	0.27‐4.2

The association of the clinical symptoms involving the nervous, hematological, and gastrointestinal systems made this clinical presentation a diagnostic challenge.

We considered a number of differential diagnosis based on the pathological changes:

1. Peripheral tetraparesis may have multiple causes: trauma, vertebral tumors, vertebral disk hernia, Guillain‐Barre syndrome, chronic degenerative neuropathy, multiple sclerosis, or other degenerative neurological diseases, as well as radiation and intoxications.

**Figure 1:**
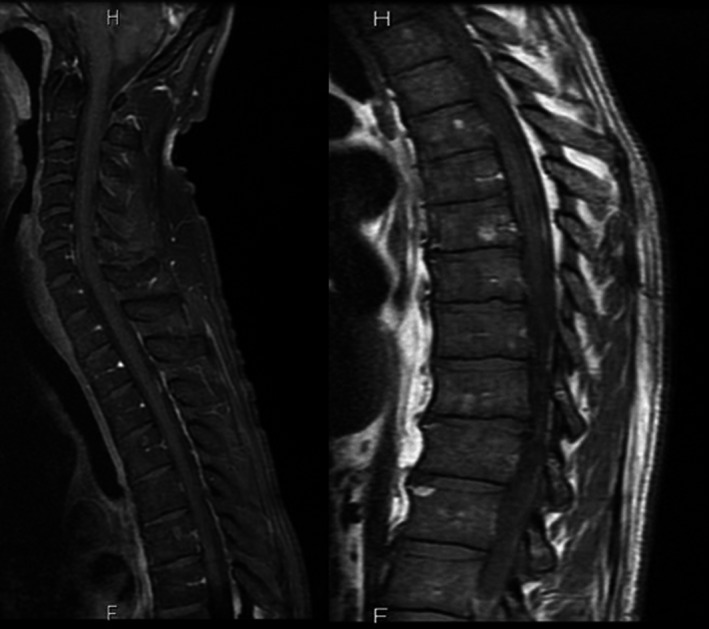
Thoracolumbar spine MRI described diffuse reduced T2‐FLAIR (Fluid‐attenuated inversion recovery) suggesting a deposit disease, as well as vertebral hemangiomas and degenerative changes

We performed a thoracolumbar and sacral spine MRI that showed no sign of tumor or trauma and described the diffuse reduced T2‐FLAIR (Fluid‐attenuated inversion recovery) at a level that is suggestive of a deposit disease (Figure 1).

- The hypochromic, microcytic anemia may have various causes but we limited ourselves to those, which combine high blood iron and ferritin levels, high saturation coefficient of transferrin and the presence of ringed sideroblasts in the bone marrow aspirate.

The presence of ringed sideroblasts in the bone marrow raises the question of a congenital anemia or of an acquired anemia induced by ethanol, other toxins, and low level of vitamin B6 or infections.

High blood iron and ferritin levels as well as the high transferrin saturation index raised the question of a congenital hemochromatosis; nevertheless, same results may be attributable to other conditions like thalassemia major, sideroblastic anemia, chronic hemolytic anemia, aplastic anemia, chronic liver disease, porphyries, elevated iron intake, or blood transfusion. We excluded hemochromatosis as the genetic test for C282Y mutation of human hemochromatosis protein (HFE) proved negative for our patient. Hemoglobin electrophoresis showed beta thalassemia minor, with a value for Hb‐A of 94% and for Hb‐A_2_ of 5.9%.

- The gastric and neurological symptoms associated with the complex hematological changes raised the diagnosis of acute intermittent porphyria. We determined the urine level of porphyrins and found an elevation of the coproporphyrins, uroporphyrins, porphobilinogen, and delta amino levulinic acid (Table 3).

**Table 3:** Values of urinary porphyrins

Test	Values	Reference intervals
Coproporphyrins (µg/24 h)	1 182	<160
Porphobilinogen (mg/24 h)	5.29	<2
Uroporphyrins (µg/24 h)	518	<60
Delta amino levulinic acid (mg/24 h)	58.6	<7

The high urinary values of different porphyrins raised the suspicion of porphyria. Acute intermittent porphyria was suggested by the elevation of the urinary level of delta amino levulinic acid and porphobilinogen. However, the normal levels of enzymatic hydroxymethylbilane synthase contradicted this diagnosis. The patient had no cutaneous manifestations seen in other forms of porphyria, which could have explained the high urinary level of porphyrins.

Other causes of increased urinary porphyrins were explored: intoxications, infectious diseases, chronic liver disease, and neoplastic disorders.

Biological tests showed high levels of blood and urinary lead of 48 µg/dL (normal, <20 µg/dL) and 290 µg/24 h (normal, <40 µg/24 h), respectively.

Our final diagnosis was nonprofessional lead intoxication complicated by peripheral tetraparesis, severe hypochromic, microcytic anemia, and beta thalassemia minor.

## 3 OUTCOME AND FOLLOW‐UP

Treatment was initiated with D‐penicillamine 1500 mg/daily, administered in two series of 10 days each, separated by a 7‐days break. Clinical and laboratory evolution was favorable. Tetraparesis gradually went into remission, gastrointestinal symptoms disappeared, anemia corrected itself, and urinary porphyrins values normalized (Table 4).

**Table 4:** Evolution of porphyrins under treatment

Test	Values	Reference intervals
Coproporphyrins (µg/24 h)	145	<160
Porfobilinogen (mg/24 h)	4.19	<2
Uroporphyrins (µg/24 h)	28.35	<60
Delta amino levulinic acid (mg/24 h)	6.67	<7

## 4 DISCUSSION

**Figure 2:**
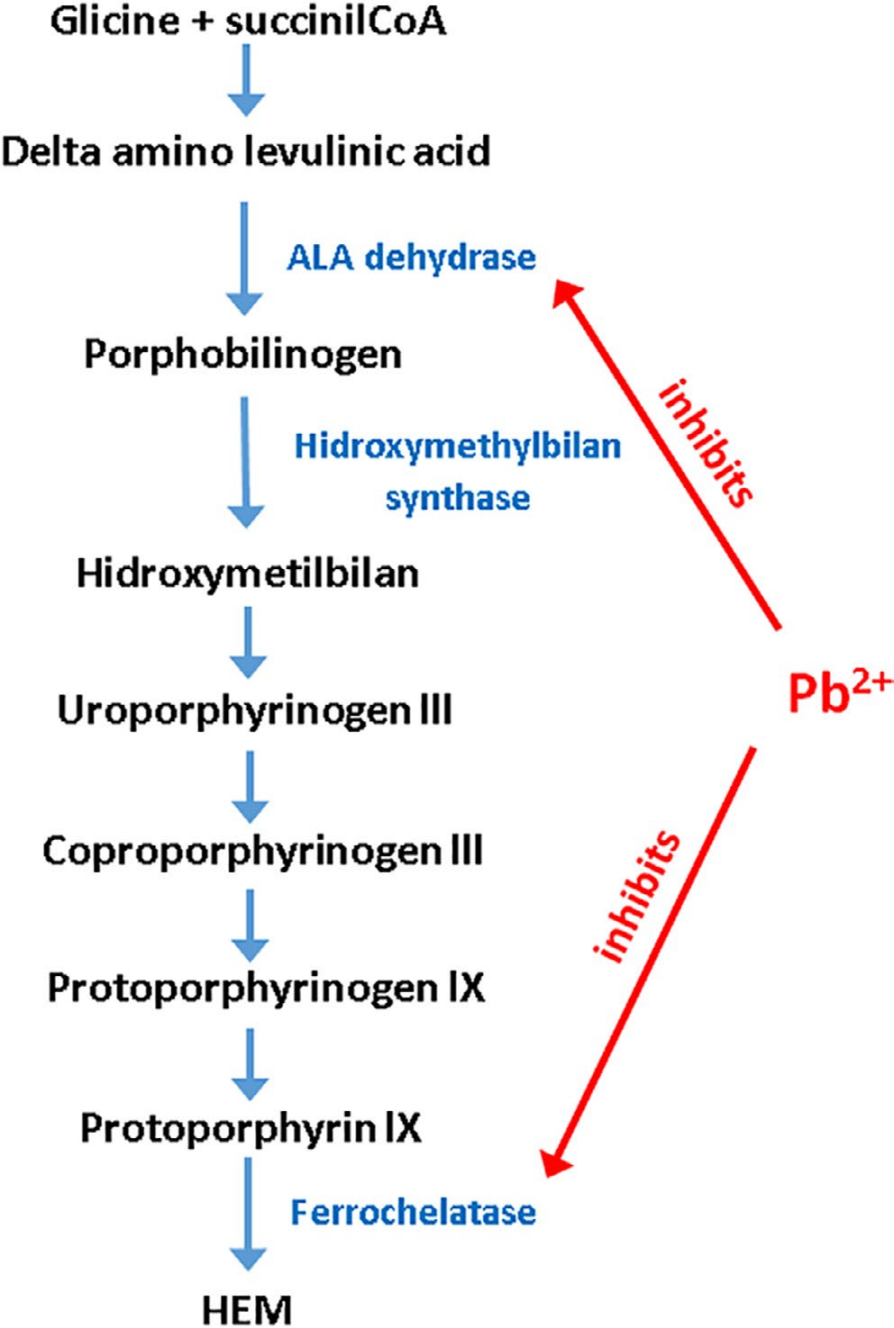
Mechanism of how lead influences heme synthesis. Pb2+ blocks the activity of aminolevulinic acid dehydrase (ALA dehydrase) and ferrochelatase involved in the synthesis of hemoglobin (see the text). In acute intermittent porphyria, the activity of hydroxymethylbilane synthase is low, as opposed to Pb2+ intoxication

Lead intoxication is responsible for over 800 000 deaths worldwide.^1^ Lead enters the organism either as an organic or inorganic (unmetabolized) form, through the respiratory and gastrointestinal tracts.^2^, ^3^ About 20%‐30% of orally ingested lead is absorbed^4^ and distributed firstly in the blood; 99% of the total blood level binds to the red cells.^4^ In the human body, 94% of lead accumulates in bones and teeth. The elimination of lead is mainly through renal excretion and through bile, with a half time of 28‐36 days.^3^ The rate of lead excretion is between 30‐200 µg/d depending on blood level.^4^ The toxicity of lead is tied to its capacity to interact with proteins through sulfhydryl, amino, phosphate, and carboxy groups and also to its capacity to mimic the actions of calcium. The high affinity for sulfhydryl groups determines lead toxicity for the synthesis of heme.^3^, ^5^ Lead inhibits ferrochelatase and ALA‐dehydratase and leads to a rise in porphyrins and delta aminolevulinic acid in urine (Figure 2). Peripheral blood smear characteristically shows red blood cells with coarse basophilic inclusions. These inclusions develop due to erythrocyte pyrimidine 5′‐nucleotidase (P5N) deficiency, which is induced by lead exposure.^6^ This enzyme dephosphorylates pyrimidine nucleoside monophosphates and maintains the correct balance into the erythrocytes between the level of pyrimidine and purine nucleotides.^6^ Acquired deficiency of P5N activity occurs after heavy metal exposure, especially lead, as well as in beta thalassemia, hence stimulating hemolysis.^6^

When its blood level exceeds 10 µg/dL, lead passes through the hematoencephalic barrier causing a loss of the links between astrocytes and the endothelial cells, as well as cerebral edema. There are also segmental demyelination and axonal degeneration in the peripheral nerves, leading to peripheral neuropathy. Lead enters different cells through the calcium (Ca^2+^) channels and binds to calmoduline, causing inflammation, muscle contraction or an inadequate immune response.^3^, ^7^

Lead intoxication can be acute or chronic. Acute intoxication involves especially neurological symptoms like paresis and muscles contractions, as well as digestive symptoms, like nausea, vomiting, abdominal pain, and constipation. In chronic lead intoxication, renal impairment and neuropsychiatric disorders are described. Specific skin changes, like blue bands bounded by black edges, named Burton lines, are visible along the gingival margin; skin is pale, described as the “plumbemic skin.” Anemia occurs in both acute and chronic forms.^7^, ^8^

In our patient the abrupt onset of clinical manifestations as well as the lack of cutaneous symptoms and renal involvement suggested an acute lead intoxication.

Disease onset with neurological and digestive symptoms associated with complex anemia raised some challenges for the differential diagnostic in terms of other possible causes.

Acute intermittent porphyria associates digestive and neurological signs, anemia and high urinary level of delta aminolevulinic acid and porphobilinogen, but there is a deficiency of the enzyme hydroxymethylbilane synthase.^10^ In our particular case, the normal blood level of hydroxymethylbilane synthase invalidated the diagnosis of an acute intermittent porphyria.

Our patient had no other cutaneous stigmata, which might evoke other types of porphyria.

Hemochromatosis was considered for the differential diagnosis because of the high levels of serum iron, high iron saturation of transferrin, and high level of ferritin, but the genetic test rejected this diagnostic.^11^ Aforementioned changes may be explained through hepatocytolysis and light hemolysis, both appearing in the context of lead intoxication.

Sideroblastic anemia was suggested by the bone marrow aspirate. A number of authors^12^, ^13^ describe the presence of ringed sideroblasts in lead poisoning. However, ringed sideroblasts may also appear in alcoholic intoxication, zinc overdose, exposure to isoniazid, antibiotics, phenacetin or busulfan, copper or vitamin B6 deficiencies.^13^, ^14^

The basophilic inclusions in erythrocytes are aggregated ribosomes and mitochondrial fragments. They may also appear in megaloblastic anemia, myelodysplastic syndromes, and hemoglobinopathies.^14^, ^15^ In lead poisoning there are coarse basophilic inclusions, in contrast to other hematological conditions.^15^

Hemoglobin electrophoresis showed that the patient had a beta thalassemia minor, which may explain the presence of target cells (codocytes) on the peripheral blood smear. Unfortunately, we obtained no data about patient's level of hemoglobin before the index event. In this case, the preexisting beta thalassemia minor represented a contributing factor for the occurrence of hemolysis and severe anemia induced by lead.

The usual form of lead‐poisoning neuropathy involves extensors of the wrist and fingers, which then spreads to other muscle groups, as blood level exceeds 40 µg/dL.^3^ Typical toxic neuropathy with distally accentuated sensory and motor involvement is less common and is rarely associated with short‐term exposure.^3^ Particularly, our patient developed peripheral tetraparesis, after a short‐term lead exposure. The source of the lead intoxication was not identified.

## 5 CONCLUSION

Despite being a rare condition, nonprofessional lead intoxication must be kept in mind in patients with ambiguous organic symptoms, which associate anemia, hemolysis, abnormal hepatic tests, neurological involvement, or abdominal manifestations. The hematological profile includes elevation of urinary porphyrins like in porphyria. However, hydroxymethylbilane synthase is not decreased in lead intoxication, as opposed to acute intermittent porphyria. The treatment with lead chelating agents may completely reverse the symptoms.

Our patient presented a distinct neurological manifestation of the acute lead poisoning and a particularly severe hemolysis, which could be explained by the association of the concomitant beta thalassemia minor. This case illustrates the difficulty to establish a correct diagnosis in the presence of multiple organ symptoms and complex laboratory findings for which it is hard to find a common denominator.

## CONFLICT OF INTEREST

None declared.

## AUTHOR CONTRIBUTION

GSG: diagnosed the patient, performed the clinical management and follow‐up of the patient, participated in the literature search, wrote the first draft and edited all versions of the manuscript. ASH: analyzed the case report, participated in the literature review, and critically evaluated the manuscript. ACDG: diagnosed the patient, performed the follow‐up of the patient, collected images and data used in the manuscript, and contributed to the editing of the manuscript. OIRZ: diagnosed the patient, provided patient care and follow‐up. ON: diagnosed the patient, provided patient care and follow‐up. ITN: advised on the discussion, made critical editions to the manuscript. AC: analyzed the case report, critically evaluated the manuscript for important intellectual content.
